# High-energy dose of therapeutic ultrasound in the treatment of patellar tendinopathy: protocol of a randomized placebo-controlled clinical trial

**DOI:** 10.1186/s12891-019-2993-2

**Published:** 2019-12-27

**Authors:** Julio Fernandes de Jesus, Tadeu Aldrovando Brihy de Albuquerque, Leandro Girardi Shimba, Flavio Fernandes Bryk, Jill Cook, Carlos Eduardo Pinfildi

**Affiliations:** 1 Human Movement Science and Rehabilitation Postgraduate Program, Universidade Federal de São Paulo – UNIFESP, campus Baixada Santista, Santos, SP 11015-029 Brazil; 2 Physical Agents and Rehabilitation Research Group GPRAE, Universidade Federal de São Paulo – UNIFESP, campus Baixada Santista, Santos, SP 11015-029 Brazil; 3 Functional Rehabilitation Specialized Group – GERF, São Paulo, SP 01239-040 Brazil; 4 Rehabilitation Sciences Program, Universidade Nove de Julho – UNINOVE, São Paulo, SP 02112-000 Brazil; 5 NANTEN Healthy and Orthopaedic Institute, São Paulo, SP 01227-000 Brazil; 6 La Trobe Sport and Exercise Medicine Research Centre, La Trobe University, Vic, Melbourne, 3086 Australia; 7 Human Movement of Science Department – Physical Therapy Course, Universidade Federal de São Paulo – UNIFESP, campus Baixada Santista, Santos, SP 11015-029 Brazil

**Keywords:** Knee, Patellar Tendinopathy, Therapeutic ultrasound, Rehabilitation

## Abstract

**Background:**

Patellar tendinopathy is an extremely debilitating condition and its treatment usually requires a combination of clinical approaches. Therapeutic ultrasound (TUS) is one of the most available electrophysical agent in rehabilitation settings; however, there is also a lack of high-quality studies that test different dosimetric aspects of TUS. Thus, the purpose of this study is to evaluate the short-, medium-, and long-term effects of the combination of high-energy TUS with a rehabilitation program for patellar tendinopathy.

**Methods:**

This will be a randomized, placebo-controlled trial with blinding of patients, assessors, and therapist. The setting is an outpatient physical therapy clinic. We will recruit 66 participants (male and female) aged between 18 and 40 years and presenting with patellar tendinopathy. A treatment combining high-energy dose TUS and a rehabilitation program for patellar tendinopathy will be delivered twice a week for 8 weeks. The control group will receive the same treatment, but with a placebo TUS. The effectiveness of the intervention will be measured at the beginning (baseline), midpoint (4 weeks), and end of treatment (8 weeks), as well as at 3- and 6-months post-treatment. Primary outcomes will be pain intensity (visual analogue scale, VAS), and VISA-P questionnaire and primary time points will be baseline (T0) and the end of the program (T2). Also, IPAQ-short form questionnaire, muscle strength (manual dynamometry), 2D kinematics, pain pressure threshold (PPT) algometry, thermography, and magnetic resonance imaging (MRI) will be collected.

**Discussion:**

TUS will be applied in an attempt to enhance the results obtained with the rehabilitation program proposed in this study, as well as stimulate some repair responses in individuals undergoing treatment for patellar tendinopathy, which in turn may optimize and improve treatment programs for patellar tendinopathy as well as to establish new guidelines for the application of TUS.

**Trial registration:**

This study was prospectively registered at April-3rd-2018 and updated at September-1st-2019 in the Brazilian Registry of Clinical Trials (REBEC) under the registration number: RBR-658n6w.

## Background

The patellar tendon is part of the knee extensor mechanism and tolerates heavy loads during lower limb movements [1]. Tendon consists of tenocytes, parallel collagen fibers, and extracellular matrix, which enable it to transfer, store, and dissipate energy [2]. Despite its structure, excessive cyclical overload can cause pathology and consequently pain and functional limitations, triggering patellar tendinopathy [3, 4].

Patellar tendinopathy is a debilitating condition that affects professional [5] and recreational [6] athletes. It is characterized by pain at the patella’s apex [7, 8] and presents with tendon pathology [9–11], reduced tendon stiffness [12], muscle recruitment inhibition, biomechanical impairments [13–15], and proprioceptive deficits [16, 17].

Generally, the rehabilitation of this condition is focused on exercise based-programs [18], especially multiphase and individualized-progressive therapeutic exercises [4]. However the management of patellar tendinopathy remains complex and difficult since a combined clinical approach that takes into account tendon pathology [19] and functional impairments [4, 20] has not yet been investigated.

Therapeutic ultrasound (TUS) is one of the most available electrophysical agent in physical therapy settings [21, 22]. Furthermore, presents some positive results about tendon pathology modulation and repair process in some experimental animal trials [23, 24]. However, the pathophysiological process of patellar tendinopathy in humans differs substantially from the experimental models [25] and making difficult translate the dosimetry from experimental animal trials to humans.

Besides that, it is still unclear which ultrasound dose is beneficial for the treatment of tendinopathy. Alexander et al. [26] showed that higher doses of ultrasound energy improved pain in individuals with shoulder calcific tendinitis. In contrast, Warden et al. [27] found that lower doses of ultrasound was no better than placebo for the treatment of individuals with patellar tendinopathy. In this context, higher doses of ultrasound might be necessary in order to produce significant improvements in patients with tendinopathy.

The etiology of patellar tendinopathy involves tendon overload [28], thus clinicians should consider general exercises to enhance kinetic chain energy dissipation and prioritizing local interventions to modify mechanical properties of the tendon [29]. Because of that, TUS dosimetry features could optimize the current clinical approach, once patelar tendinopathy has a potential to become recalcitrant [30]. However, there are no studies that have tested high-energy doses of TUS combined with exercise-based rehabilitation programs for patellar tendinopathy.

Thus, this placebo-controlled clinical trial aims to evaluate pain, motor function, and muscle strength of the lower limbs of individuals with patellar tendinopathy who complete a rehabilitation program combined with high-energy TUS.

## Methods

### Aim

The aim is to compare the effects in short-, medium-, and long-term of high-energy TUS or its placebo with a rehabilitation program for patellar tendinopathy.

### Study design

This is a protocol of an ongoing randomized, placebo-controlled trial with blinding of patients, assessors, and therapist.

### Study settings

The participants will be treated in an outpatient physical therapy clinic from São Paulo, Brazil. This project was approved by the Research Ethics Committee of the Federal University of São Paulo (UNIFESP) under registration number 2.351.182. To participate in this study all participants will give consent and sign the informed Consent Form  (Additional file 2), before baseline assessment. It was also prospectively registered at April-3rd-2018 and updated at September-1st-2019 in the Brazilian Registry of Clinical Trials (REBEC) under the registration number: RBR-658n6w. If there is a need to change the protocol, the change will be immediately reported to the Research Ethics Committee and carried out at the registration site.

There aren’t any publications or submissions containing any of the results of this study.

### Participants

We will recruit individuals of both sexes who are seeking treatment for patellar tendinopathy in primary and secondary health services (new presentations), aged between 18 and 40 years-old, with localized pain (pain map will be marked by the participants) at the inferior patellar pole/superior aspect of patellar tendon with load (single leg decline squat) [31], pain when jumping/landing, running or abruptly changing direction, and with a score of less than 80 on the Victorian Institute of Sports Assessment - Patella (VISA-P) questionnaire [32–34].

Participants will be excluded if they present a history of nervous and/or musculoskeletal injuries in the low back spine and/or lower limbs in the last 6 months [35], signs and/or symptoms of other diseases and/or another disorders affecting the knees [27], including systemic and rheumatic diseases and/or pre-existing dysfunctions (clinically evaluated and detected) [36], previous history of knee or patellar tendon surgery [27, 37], use of intra-articular medications in the knee in the last 6 months [27], or pregnancy. A single physician – who consults at primary and secondary health services will standardize recruitment and determine eligibility, based on clinical history testing, along with imaging examination of potential participants.

### Randomization and masking

All participants will be informed about the study procedures and then they will be randomly allocated to one of the intervention groups (active TUS or placebo TUS). The researcher not involved with the data collection will allocate the participant using numbers (1 or 2) placed according to the sequence presented in the plan and sealed in opaque envelopes without stratifications or covariates consideration. The randomization sequence was created at http://www.randomization.com, in a single block with two distinct labels: 1 and 2 (referring to the two groups of this study).

Participants and the therapist will be blinded throughout the treatment. To ensure blinding, two identical TUS machines will be used, however one will have its software modified by the manufacturer to simulate sound wave emission (30 s of stimulation followed by an undetectable pause in the sound wave emission) and only a researcher who is not involved in collecting data knows if the equipment is active or placebo. Furthermore, and to keep the therapist blinded, the sessions outcomes: visual analogue scale (VAS) – VAS-usual and -irritative, pain pressure threshold (PPT)-algometry, and thermography will be collected by an assessor (not the therapist) who will be unaware of each participant’s group allocation; and the treatment room will be set up by a research assistant with only one TUS equipment (in agreement with participant group allocation: active or placebo). The participants will be informed before the treatment about the characteristics of the rehabilitation program and asked to not discuss the TUS application with the therapist and assessor researchers.

### Intervention

Participants will take part in the exercise program (Tables 1 and 2) and TUS application (active or placebo). If there are bilateral complaints, the most symptomatic knee will receive TUS but both lower limbs will receive exercises.

**Table 1:** Exercises program - phases I and II

Phase (weeks)	Exercises	Weight	Series
I (0-2nd weeks) 4 sessions	Gluteal Muscles - *Fire Hydrant*	Intermediary elastic resistance (green)	Isometric (1st wk) - 3 × 45” Isotonic (2nd wk) - 3 × 15
	Gluteal Muscles - *Clam Shell*	Intermediary elastic resistance (green)	Isometric (1st wk) - 3 × 45” Isotonic (2nd wk) - 3 × 15
	Gluteus Medius - Lateral straight leg raise	No extra-weight (1st wk) - lower limb weight 0,5 kg (2nd wk)	Isotonic - 3 × 15
	Quadriceps 1 - Unilateral Leg Extension Machine *Isometric ROM → 60° flexion* *Isotonic ROM → 10°-60° flexion*	70% of MVIC (adjusted for both lower limbs)	Isometric (1st wk) - 3 × 45” Mixed (2nd wk) - isometric 1 × 45” + isotonic 3 × 15
	Quadriceps 2 - bipodal *Spanish Squat* *ROM → 70°-90° flexion*	Body weight	Isometric (1st wk) - 3 × 45”
	Quadriceps 3 - Unipodal Squats (sit/stand bench)	Body weight	Isotonic (2nd wk) - 3 × 10
	Triceps Surae - Bipodal Heel Elevation	Body weight	Isotonic - 3 × 20
II (3rd-4th weeks) 4 sessions	Gluteal Muscles - *Fire Hydrant*	Intermediary elastic resistance (blue)	Isotonic - 3 × 15
	Gluteal Muscles - *Clam Shell*	Intermediary elastic resistance (blue)	Isotonic - 3 × 15
	Gluteus Medius - Lateral straight leg raise	1,0 kg (3rd wk) 1,5 kg (4th wk)	Isotonic - 3 × 15
	Quadriceps 1 - Unilateral Leg Extension Machine *Isometric ROM → 60° flexion* *Isotonic ROM → 10°-60° flexion*	80% of MVIC (adjusted for both lower limbs)	Mixed - isometric 1 × 45” + isotonic 3 × 15
	Quadriceps 3 - Unipodal Squats (sit/stand bench)	Body weight	Isotonic - 3 × 12
	Quadriceps 4 - *Single Leg Decline Squat* *only eccentric phase	Body weight	Isotonic - 3 × 10
	Triceps Surae - Bipodal Heel Elevation	4 kg (diving belt weight)	Isotonic - 3 × 20

*wk* week; *ROM* Range of motion; *MVIC* Maximal voluntary isometric contractions

**Table 2:** Exercises program - phases III and IV

Phase (weeks)	Exercises	Weight	Series
III (5th–6th weeks) 4 sessions	Gluteal Muscles - *Fire Hydrant*	Intermediary elastic resistance (purple)	Isotonic - 3 × 12
	Gluteal Muscles - *Clam Shell*	Intermediary elastic resistance (purple)	Isotonic - 3 × 12
	Gluteus Medius - Lateral straight leg raise	2,0 kg (5th wk) 2,5 kg (6th wk)	Isotonic - 3 × 12
	Quadriceps 1 - Unilateral Leg Extension Machine *Isometric ROM → 60° flexion* *Isotonic ROM → 10°-60° flexion*	90% of MVIC (adjusted for both lower limbs)	Mixed - isometric 1 × 45” + isotonic 3 × 12
	Quadriceps 4 - *Single Leg Decline Squat* *only eccentric phase	Body weight	Isotonic - 3 × 12
	Quadriceps 5 - *Walk Lunge*	10 kg (5 kg in each hand)	3 × 10 steps
	Triceps Surae - Step Bipodal Heel Elevation	6 kg (diving belt weight)	Isotonic - 3 × 15
IV (7th–8th weeks) 4 sessions	Gluteal Muscles - *Fire Hydrant*	Intermediary elastic resistance (gray)	Isotonic - 3 × 10
	Gluteal Muscles - *Clam Shell*	Intermediary elastic resistance (gray)	Isotonic - 3 × 10
	Gluteus Medius - Lateral straight leg raise	3,0 kg	Isotonic - 3 × 10
	Quadriceps 1 - Unilateral Leg Extension Machine *Isometric ROM → 60° flexion* *Isotonic ROM → 10°-60° flexion*	100% of MVIC (adjusted for both lower limbs)	Mixed - isometric 1 × 45” + isotonic 3 × 10
	Quadriceps 4 - *Single Leg Decline Squat* *only eccentric phase	Body weight	Isotonic - 3 × 15
	Quadriceps 5 - *Walk Lunge*	10 kg (5 kg in each hand)	3 × 10 steps
	Triceps Surae - Step Unipodal Heel Elevation	8 kg (diving belt weight)	Isotonic - 3 × 15

*wk* week; *ROM* Range of motion; *MVIC* Maximal voluntary isometric contractions

The TUS dosimetric settings [26, 38, 39] are described in Table 3 and the applications will be carried out twice a week during the treatment period (8 weeks - 16 sessions) and always at the beginning of the session (before exercise). Individuals will be placed in supine during the application with the knee flexed at approximately 20° and stabilized by a positioning cushion.

**Table 3:** Therapeutic ultrasound (TUS) dosimetric parameters

Parameters	Adjusts
Frequency	1 MHz
Mode	Continuous
Effective radiation area (ERA)	7 cm^2^
Spatial average – Temporal peak (SATP)	1,2 W/cm^2^
Time (t)	8 min
Energy (E)	4032 J / per application
Beam non-uniformity ratio (BNR)	3:1
Application speed	Slow (± 4 cm/s)

**Fig. 1:**
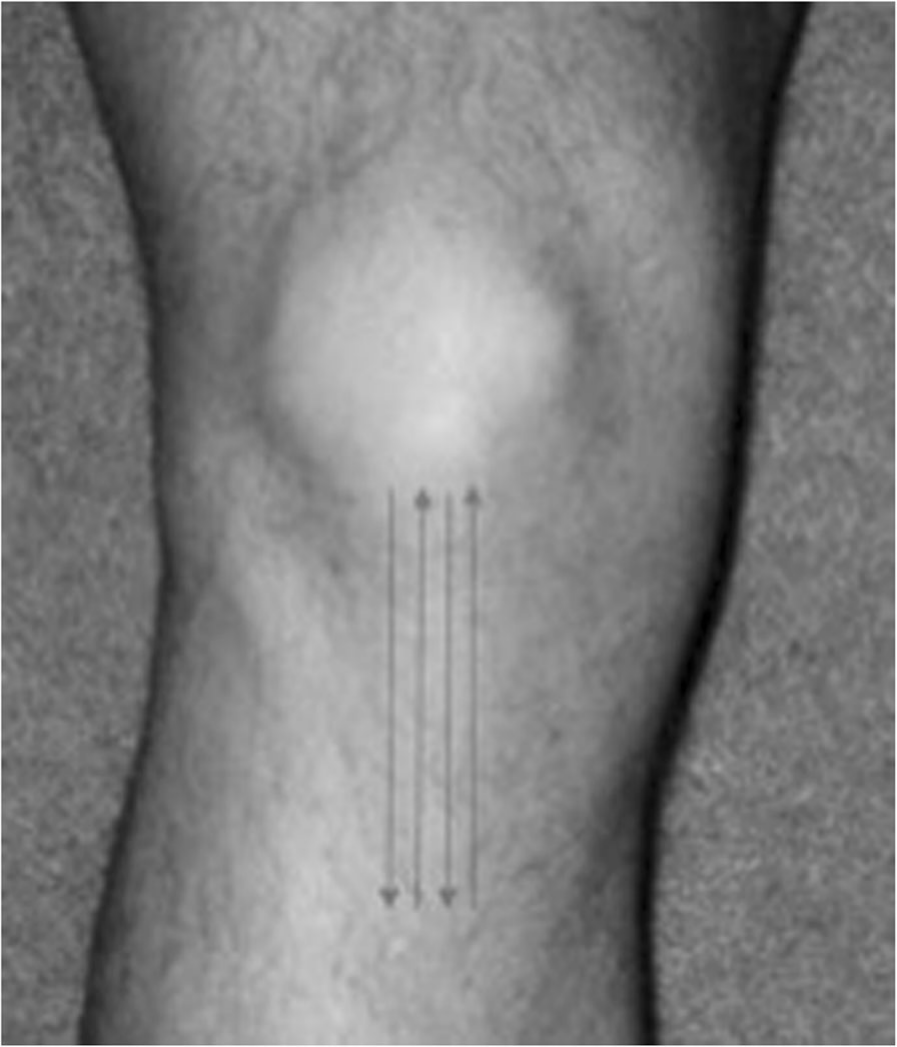
Local of TUS application (patellar tendon)

TUS will be applied in the patellar tendon region (Fig. 1), with full contact between the TUS transducer and the treatment area. The gel will be used as the conduction agent and circular movements of the head device will be performed at a slow speed, approximately 4 cm/s.

A program of therapeutic exercises was developed based on clinical features [40–42] and specific strategies for patellar tendinopathy management [4, 43] and also in similar exercises [44, 45] and protocols applied in other overload conditions [46]. This program will be delivered in four phases, each one containing 2 weeks duration [20, 35, 47] (Tables 1 and 2) and the standardized sessions will be performed in individually and will be supervised by the same physical therapist that is also responsible for all TUS applications. The participants will be instructed not to use other interventions, but will be encouraged to stay active in their physical activities or sports during this program, however respecting their level of function and pain. This information will be given to the participants at the initial examination.

### Outcome measure

Pain intensity will be measured using the Visual Analogue Scale (VAS) [48, 49]. This outcome will be always based on routinely presented pain (VAS-usual) and pain during the eccentric decline squat (VAS-irritative at pre-TUS and post-TUS) [27, 50]. Pain, dysfunction and the level of physical activity will be measured with the VISA-P questionnaire [34] and the International Physical Activity Questionnaire-Short Form (IPAQ-short form) [51], respectively.

The trunk and lower limbs kinematics will be assessed with two-dimensional (2D) kinematic analysis, once patellar tendinopathy may cause kinematic changes at hip [33, 52], knee [53, 54] and foot [53, 54] levels during landings the 2D-kinematics analysis will be applied in attempt to detect lower limb and trunk kinematic patterns on different types and intensities of landing tasks in both groups.

The acquisition of these images will be carried out with two cameras Optitrack (NaturalPoint Inc., Corvallis, OR, USA) with 0.3 MP resolution and capture rate of 100 FPS, two cameras (color video capture) Logitech - Model: C920 (Logitech, Newark, CA, USA) with 3 MP resolution and capture rate of 30 FPS and with the software myoVIDEO - MR3 3.6.43 (Noraxon USA Inc., Scottsdale, AZ, USA). Reflective markers will be applied on the acromion bilaterally, manubrium, xiphoid process, antero-superior iliac spines, patellar bases, ankle joint lines, major trochanters, lateral femoral condyles of the knees, lateral malleoli, intermediate third of the fibula diaphysis, and on the tuberosity of the fifth metatarsals [55, 56].

The following movements will be verified and quantified in the frontal plane: trunk inclination, pelvic tilt, hip adduction, and knee valgus; and in the sagittal plane: trunk flexion, hip flexion, knee flexion, and ankle dorsiflexion. The time to acquire the peak knee flexion will also be quantified. The motion cameras for the frontal and sagittal planes will be placed 3.5 m and 3.0 m from the participant, respectively, for data collection on Single Leg Squat (SLS) [55, 57] and Drop Vertical Jump Test - Double and Single Leg (DVJ-DL and DVJ-SL) [55, 58]. For the Single Hop Test for Distance (SHTD) [59], the cameras will be placed 3.7 m and 3.0 m from the participant for frontal and sagittal plane data collection, respectively. The motion analyses will be performed at peak knee flexion, as this moment will be the reference for all measurements.

The strength (maximum voluntary isometric contraction, MVIC) of the gluteus maximus [42, 60] (hip extension), gluteus medius [61] (hip abduction), quadriceps femoris [59] (knee extension), and triceps surae [42] (ankle plantar flexion) will be assessed with a handheld dynamometer (Manual Muscle Tester, model 01163, Lafayette Instrument Company, Lafayette, IN, USA) [62]. A nylon belt will be positioned perpendicular to the dynamometer during the tests in order to stabilize the dynamometer and to resist the force generated by the participant. Two 5-s MVIC will be performed with a 30-s interval between contractions [59]. The average strength of each muscle - obtained in kilogram/force (KgF) - will be normalized by the body weight of each participant, using the following formula: (muscle strength kg/ body weight kg) X 100 [59].

Pain pressure threshold (PPT)-algometry (PPT – algometer model: DD-2000, Instrutherm, São Paulo, SP, Brazil) of the patellar tendons will be performed at T0, T1 and T2 evaluations time-points and at every session – before and after the TUS application (± 8′ interval between PPT evaluation). For this measurement, the participant will be positioned in supine, with the knee flexed (± 20°) and stabilized with a positioning cushion and as soon as the individual experienced a painful/discomfort sensation he/she will say “stop”; the PPT algometer will be immediately released and the force (in Newtons) will be read from the display. The data will be collected twice (the average will be used) directly on the patellar tendon (just below the apex of the patella) [63].

There is more specific information about 2D kinematic analysis, muscle strength dynamometer and PPT algometry at Additional file 1.

Thermography of the patellar tendons will be assessed with the thermal camera FLIR C2 (FLIR Systems Inc., Danderyd, Sweden), which has a resolution of 320X240 pixels and in accordance with international recommendations [64]. Two images will be captured, and the average of the two images will be used. Before collecting data on thermography, participants will remain for 10 min in a room at ambient temperature. The camera will be positioned perpendicular to the patellar tendon at a distance of approximately 30 cm [65–68].

Possible tissue changes of the patellar tendons or adverse events will be assessed by magnetic resonance imaging (MRI) at two separate time points: before (T0; initial MRI) and after the treatment program (T2-final MRI). The images will be acquired in a magnetic field of 1.5 T (high field). The MRI protocol will consist of proton-density (PD) T2-weighted fast spin-echo (FSE) sequences acquired in the coronal, axial, and sagittal planes, with pulse sequence and cuts of 3 mm thickness, 0.3 mm inter-cut space, repetition time (TR) of 3760 ms, echo time (TE) of 16.25 ms and 97.54 ms, respectively, without averages, field of view (FOV) of 150 X 150 X 106 mm, 32 cuts with acquisition matrix of 256 X 256, resulting in 0.586 mm of plane resolution [69, 70]. The following characteristics will be assessed: intra-tendinous signal, involvement to the retropatellar region (Hoffa’s fatpad), tendon volume, the area of the maximum transverse section of the tendon, maximum antero-posterior tendon diameter, and signal intensity [70, 71]. A physician will perform the MRI assessments with clinical experience in imaging analysis, with no knowledge of group allocation or MRI order (initial or final).

### Data collection

After recruitment and before the beginning of treatments, demographic/personal data, clinical history (duration of symptom), and patellar tendon morphological characteristics (initial magnetic resonance imaging - MRI) will be collected.

The outcomes of this study (Table 4) will be collected in five time-points: before starting the program (baseline – T0), in the middle (after 4 weeks/8 sessions – T1), in the end (after 8 weeks/16 sessions – T2), three and 6 months after the end of the program (follow-ups T3 and T4, respectively). The T3 and T4 data collection will be performed by mobile text messages, telephone contact or mail (depending on participant preference). However, the primary time points will be baseline (T0) and the end of the program (T2).

**Table 4:** Outcome measured and time-points

Outcomes	Data collection time-points					
	T0^a^	All sessions	T1	T2^a^	T3	T4
Primary						
1. VAS	X	X	X	X		
2. VISA-P	X		X	X	X	X
Secondary						
3. IPAQ-short form	X		X	X	X	X
4. PPT algometry	X	X	X	X		
5. Thermography	X	X	X	X		
6. Muscle strength	X		X	X		
7. 2D kinematics	X		X	X		
8. MRI	X			X		

^a^ Primary time-points

*VAS* Visual analogue scale; *VISA-P* Victorian Institute of Sports Assessment-Patella; *IPAQ-short form* International Physical Activity Questionnaire-Short Form; *PPT* Pain Pressure Threshold; *2D kinematics* two-dimensional kinematic analysis; *MRI* Magnetic resonance images

Beyond that, in all sessions will be collected VAS[48, 49]-usual, VAS-irritative: pre- and post-TUS [4]; and thermography: pre-TUS, post-TUS, pre-quadriceps exercises, and at the end of the session. The MRI will only be acquired at the beginning and at the end of treatment program.

**Fig. 2:**
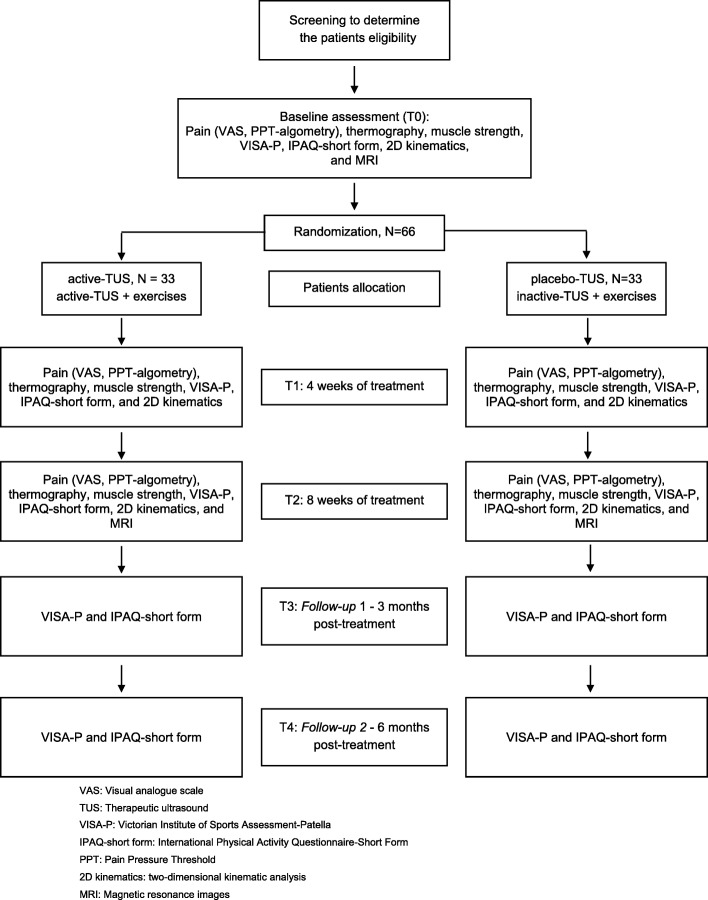
CONSORT study flowchart

An assessor who will be unaware of each participant’s group allocation will collect all these outcomes. Figure 2 shows the flowchart with a summary of experimental procedures and the participation of individuals, according to the Consolidated Standards of Reporting Trials (CONSORT) [72].

### Data analysis, monitoring, and auditing

The sample size was calculated using the primary outcome variables: VAS and VISA-P questionnaire. A higher number of participants per group was required when the calculation was performed using data from the VISA-P questionnaire. For the calculation, the minimum detectable difference (MDD) considered was 12.2 points [34], with an average of the standard deviations of 16.2 points [27], resulting in 28 participants per group for an α-error of 0.05 and a β-error of 80%. Thus, 33 participants per group (assuming 15% of possible sample losses) will be selected.

The head-coordinator (CEP) of the present study will be the responsible for the monitoring and auditing process of all stages related to this clinical trial, as well as be responsible for terminate it. These processes will be performed at regular times by personal visiting of data setting collection and checking the data bank archive, and if some irregularity or adverse event is detected; the trial will be interrupted.

All assessment forms will be scanned and stored in a safe place, and the data tabulation will be stored on the study’s computer and on pen drives to avoid losses. The participants will be coded by numbers to maintain the confidentiality of their personal data, which will not be disclosed. One of the researchers (CEP) will certify the data collection stage. The final results of the study will only be revealed upon completion of all follow-ups, and only the authors of the research will have access to the data prior to publication in a peer-review journal. The data will be entered twice to avoid tabulation errors, and the statistician will receive the coded data and will be blind to group allocation.

The statistical analysis will be conducted following the principles of intention-to-treat analysis [73]. The normality of the data will be tested with visual inspection of histograms and the characterization of participants will be calculated using descriptive statistics. Improvements from baseline (T0) until the rehabilitation program final time-point (T2) will be assessed with a mixed model ANOVA (split-plot ANOVA). The differences between groups (treatment effects) and their respective confidence intervals (95% CI) will be calculated for all outcomes, using mixed linear models for the interaction of groups by time. These analyses will be carried out using statistical software (Statistical Package for Social Sciences (SPSS) version 15.0).

## Discussion

This randomized, placebo-controlled, blinded clinical trial aims to examine the effectiveness of the combination of high-energy dose of TUS and a multiphase exercise rehabilitation program for patellar tendinopathy and determine whether TUS influences pain modulation and other outcomes such as muscle strength, lower limb motor function and morphological tendon characteristics.

Patellar tendinopathy is a condition with difficult clinical management due to its multifactorial etiology [74, 75]. Individuals suffering from patellar tendinopathy present with structural, biochemical and consequently functional changes that lead to load absorption and propagation deficits, and pain [76]. In addition, these changes hamper tissue repair and tend to cause perpetuation of the condition [28]. The outcomes chosen for this study were based on these changes with the aim of understanding the possible pathways of TUS in the modulations of the signs and symptoms in individuals with patellar tendinopathy.

Therapeutic exercises are the most researched interventions for the treatment of patellar tendinopathy, especially the eccentric decline squat, as it loads the injured patellar tendon [29]. It is possible that pain reduction improves muscle recruitment and consequently modulates lower limb biomechanics [77], promoting functional optimizations. However, therapeutic exercises in isolation do not always solve all of the complex symptoms of these patients, especially very painful symptoms and those of high-performance athletes during sports seasons [31].

Thus, TUS may enhance the results obtained with the rehabilitation program proposed in this study, as well as stimulate some repair responses [23, 24] in individuals undergoing treatment for patellar tendinopathy. The current literature shows increases in patellar tendon temperature after the application of TUS [78], an effect that could raise the pain threshold [79] and improve collagen extensibility [80]. These effects could favor the viability of the proposed exercises, optimizing the results. The add-on (chronic) effect promoted by high-energy TUS [81] could also influence the tissue structure of the tendons, making them more resistant and better able to absorb and dissipate energy, thus improving function in individuals with patellar tendinopathy.

Although there is evidence in the literature showing a lack of clinical benefits from TUS in the treatment of patellar tendinopathy [27], there is a lack of research with good methodological design testing the use of high-energy TUS combined with rehabilitation programs for this clinical condition. In addition, the lack of positive TUS results for the treatment of the most diverse conditions has been related to dosimetric errors [82].

In this context, Alexander et al. [26] demonstrated that doses of 2250 J (J) of energy (per treatment session) promoted improvement in pain in individuals with painful shoulders, while doses of 750 J or less rarely generated positive results. The treatment of elbow tendinopathy and calcaneal tendinopathy with a TUS dose of 18,250 J promoted significant improvement in pain and function [83]. Similarly, the application of doses of 1290 J, 2580 J, and 11,250 J was effective for the treatment of calcific tendonitis of the shoulder, resulting in lower pain rates, improved motor function, and regression of pathological calcification [38, 39].

Due to these characteristics, the TUS dose will be 4032 J per application combined with a rehabilitation program based on current and scientifically developed clinical guidelines [4, 40–42]. The dosage for the application of TUS is one of the most difficult factors to translate from animal studies to the clinical setting. The results from the animal studies open the way for the investigation of the effects of TUS in humans; however, the dosimetric factor is complex and there is no direct correlation between animals and humans. Therefore, the energy parameter (J) is being used as a facilitator to the replication of these parameters.

The current study has a high methodological quality, as it is a prospective, randomized, and controlled clinical trial. The blinding of assessors, therapists, and patients, who will not have access to the characteristics of the interventions during the study, yields greater methodological relevance.

Finally, the sample size was calculated with the purpose of providing adequate statistical power and sensitivity to detect differences in the primary and secondary outcomes. Therefore, the design features of the current study allow a relevant contribution to evidence-based practice, particularly as concerns the clinical management of patellar tendinopathy with the use of TUS.

## Supplementary information

**Additional file 1.** Additional informations of 2D kinematic analysis, muscle strength dynamometer, and PPT-algometry.

**Additional file 2.** Consent statement.

## Data Availability

The datasets that will be used and/or analyzed during the current study will be available from the corresponding author on reasonable request.

## Abbreviations

2D: Two-dimensional
DVJ-DL: Drop Vertical Jump Test - Double Leg
DVJ-SL: Drop Vertical Jump Test - Single Leg
FOV: Field of view
FSE: Fast spin-echo
ICC: Intraclass correlation coefficient
IPAQ-short form: International Physical Activity Questionnaire-Short Form
J: Joules
KgF: Kilogram/force
MDD: Minimum detectable difference
MRI: Magnetic resonance images
MVIC: Maximal voluntary isometric contractions
PD: Proton-density
PPT: Pain pressure threshold
REBEC: Brazilian Registry of Clinical Trials
SHTD: Single Hop Test for Distance
SLS: Single Leg Squat
SPSS: Statistical Package for Social Sciences
TE: Echo time
TR: Repetition time
TUS: Therapeutic ultrasound
VAS: Visual analogue scale
VISA-P: Victorian Institute of Sports Assessment Questionnaire, Patellar
