# Supplementary material for: High-energy dose of therapeutic ultrasound in the treatment of patellar tendinopathy: protocol of a randomized placebo-controlled clinical trial
Source: BMC Musculoskelet Disord. 2019 Dec 27;20:624. doi: 10.1186/s12891-019-2993-2 (PMC6933732; doi:10.1186/s12891-019-2993-2)
Supplement: Supplementary file 1 — Additional file 1. Additional informations of 2D kinematic analysis, muscle strength dynamometer, and PPT-algometry. [file 12891_2019_2993_MOESM1_ESM.pdf]

ADDITIONAL FILE 1

- 2D kinematics analysis

The biomechanics analyzes will be performed through the execution of the following functional tests:

- Single Leg Squat (SLS): The participants will be placed on a step (length: 65 cm X height: 18 cm X width: 25 cm) and instructed to adopt one-legged support, keep the arms crossed behind the trunk and perform three squats up to the maximum of knee flexion (lower limb over the step) that they can. The contralateral limb will always remain hanging out of the previous anterior step limits. The cameras that will capture the movements performed in the frontal and sagittal plane, will be at 3.5 m and 3.0 m distance - respectively - of the participant in his position of beginning of the test[1, 2].
- Drop Vertical Jump Test - Double and Single Leg (DVJ-DL e DVJ-SL): The participants will be positioned on the same step mentioned above and will be guided to make a small frontal jump, just to loose contact with the step; followed by a maximum vertical jump (as high as possible) and ending with the landing. This test will be performed initially with bipodal (DVJ-DL) and then unipodal (DVJ-SL); when the DVJ-SL is performed, all stages of the test will be carried out with unipodal weight-bearing. The cameras that will capture the movements performed in the frontal and sagittal plane, will be at 3.5 m and 3.0 m distance - respectively - of the participant in his position of beginning of the test[1, 3].
- Single Hop Test for Distance (SHTD): The participants will be positioned at a starting point demarcated on the ground, will adopt unipodal weight-bearing,

keep the upper limbs crossed behind the trunk. Finally, they will be encouraged to jump as far as possible from this starting point, provided a landing and remain stable on the assessed lower limb for at least two seconds. Two official attempts will be made for each evaluated lower limb (the jumped distance will be represented by the simple arithmetic mean of these attempts); however, individuals will be able to perform as many jumps as they deem necessary to become familiar with this test. Individuals will be barefoot to perform this test and will always be measured at hallux-hallux distance (initial-final). The cameras that will capture the movements performed in the frontal and sagittal planes, will be at 3.7 m and 3.0 m distance - respectively - of the participant in his position of beginning of the test[4].

- Muscle strength dynamometer

The participants will be instructed to perform their maximum strength during the tests prior to the strength assessment. However, a voluntary submaximal isometric contraction will be performed initially to familiarize the individual with each test, followed by two maximal voluntary isometric contractions (MVIC) lasting 5 seconds each and 30 seconds intervals between the tests. If the values show a difference greater than 10%, a new measurement will be performed (after the participant rests for 2 minutes) and the final final result will be calculated by the simple arithmetic mean[4].

Prior to the start of this data collection, a pilot study was conducted to evaluate the intra-class correlation coefficient (ICC) of the evaluator responsible for collecting the gluteus maximus, gluteus medius, quadriceps femoris, and triceps surae manual dynamometer. In this way, five healthy individuals who did

not practice regular physical activities (10 lower limbs: 5 dominant and 5 non-dominant) were evaluated. These participants were tested in the same way as the protocol described above, with a 7-day interval between the evaluations and the results obtained showed the following correlations: excellent for gluteus maximus (ICC = 0.94), excellent for gluteus medius (ICC = 0.95), excellent for quadriceps femoris (ICC = 0.97) and excellent for triceps surae (ICC = 0.80). The positions adopted by the individuals for the accomplishment of the strength evaluations will be the following ones:

- Gluteus maximus: The participants will be positioned at ventral decubitus, with hips in neutral position and arms aside of the trunk. The evaluated limb knee will be flexed at 90° and the contralateral knee will be kept in neutral. The dynamometer will be positioned on the posterior surface of the thigh - approximately 5 cm above the popliteal fossa - and a nylon belt will be used to stabilize the lumbar region and avoid compensations during the evaluations[5, 6].
- Gluteus medius: The participants will be positioned at lateral decubitus, with the lower limb to be assessed at approximately 20° of abduction, 10° of extension and neutral rotation, the knee will always be held in extension. The dynamometer will be positioned on the lateral side of the knee (lateral femoral condyle) of the assessed limb[7].
- Quadriceps femoris: The participants will be seated in an extensor chair, hips at 90° flexion and 0° rotation, knees at 60° flexion. A three-point nylon belt will be used to stabilize the trunk and the hips. The dynamometer will be positioned in the anterior region of the leg, approximately 2.5 cm above the medial malleolus[4].

- Triceps surae: The participants will be positioned at ventral decubitus with knees in extension. The dynamometer will be positioned on the forefoot plantar surface of the evaluated limb (hanging out of the therapeutic bench limits)[6].

• Pain pressure threshold algometry

The pain pressure threshold (PPT) algometry of the patellar tendons (painful site) will be performed in all sessions, before (pre-UST) and just after the application of therapeutic ultrasound (post-UST) and also in the evaluations: T0, T1 and T2. Prior to the start of the present study, a pilot study was conducted to evaluate the reproducibility (intraclass correlation coefficient - ICC) and validity of the evaluator responsible for collecting the data with the pressure algometer. In this way, five healthy individuals who did not practice regular physical activities (10 lower limbs: 5 dominant and 5 non-dominant) were evaluated. These participants were tested in the same way as the protocol described at the manuscript, with a 7-day interval between the evaluations and the results obtained demonstrated good reproducibility (ICC = 0.73).

REFERENCES

1. Dingenen B, Malfait B, Vanrenterghem J, Verschueren SM, Staes FF: **The reliability and validity of the measurement of lateral trunk motion in two-dimensional video analysis during unipodal functional screening tests in elite female athletes**. *Phys Ther Sport* 2014, **15**(2):117-123.

2. Jones D, Tillman SM, Tofte K, Mizner RL, Greenberg S, Moser MW, Chmielewski TL: **Observational ratings of frontal plane knee position are related to the frontal plane projection angle but not the knee abduction angle during a step-down task**. *J Orthop Sports Phys Ther* 2014, **44**(12):973-978.
3. Nilstad A, Andersen TE, Kristianslund E, Bahr R, Myklebust G, Steffen K, Krosshaug T: **Physiotherapists can identify female football players with high knee valgus angles during vertical drop jumps using real-time observational screening**. *J Orthop Sports Phys Ther* 2014, **44**(5):358-365.
4. Fernandes de Jesus J, de Almeida Novello A, Bezerra Nakaoka G, Curcio Dos Reis A, Fukuda TY, Fernandes Bryk F: **Kinesio taping effect on quadriceps strength and lower limb function of healthy individuals: A blinded, controlled, randomized, clinical trial**. *Phys Ther Sport* 2016, **18**:27-31.
5. Jesus JFd, Bryk FF, Moreira VC, Nakaoka GB, Reis ACd, Lucareli PRG: **Gluteus Maximus inhibition in proximal hamstring tendinopathy**. *MedicalExpress* 2015, **2**.
6. Scattone Silva R, Nakagawa TH, Ferreira AL, Garcia LC, Santos JE, Serrao FV: **Lower limb strength and flexibility in athletes with and without patellar tendinopathy**. *Phys Ther Sport* 2016, **20**:19-25.
7. Magalhaes E, Fukuda TY, Sacramento SN, Forgas A, Cohen M, Abdalla RJ: **A comparison of hip strength between sedentary females with and without patellofemoral pain syndrome**. *J Orthop Sports Phys Ther* 2010, **40**(10):641-647.
