# Supplementary material for: High-energy dose of therapeutic ultrasound in the treatment of patellar tendinopathy: protocol of a randomized placebo-controlled clinical trial
Source: BMC Musculoskelet Disord. 2019 Dec 27;20:624. doi: 10.1186/s12891-019-2993-2 (PMC6933732; doi:10.1186/s12891-019-2993-2)
Supplement: Supplementary file 2 — Additional file 2. Consent statement. [file 12891_2019_2993_MOESM2_ESM.pdf]

TERMO DE CONSENTIMENTO LIVRE E ESCLARECIDO

Ministério da Educação
Universidade Federal de São Paulo
Campus Baixada Santista
Departamento de Ciências do Movimento Humano

HIGH-ENERGY DOSE OF THERAPEUTIC ULTRASOUND IN THE TREATMENT OF PATELLAR TENDINOPATHY: A RANDOMIZED PLACEBO-CONTROLLED CLINICAL TRIAL

This information is being provided for voluntary participation in this study, which aims to investigate the effects of high dose therapeutic ultrasound associated with exercises to treat pain in the knee (patellar tendon). We believe that the therapeutic ultrasound applied with high energy dose in the patellar tendon can alleviate their pain and improve their performance in the proposed exercises and thus improve the obtained result. To achieve our goals you will be subject to the following protocol:

1. You will be submitted to the application of therapeutic ultrasound in the complainant patellar tendon. Ultrasound consists of an emission of sound waves capable of interacting with the tendinous cells and stimulating their functioning. The ultrasound is applied while you will be in the lying position, through contact with the skin and by a qualified physiotherapist. The gel (water-based) will be used as a means of contact between the therapeutic ultrasound and the skin.

2. After applying the therapeutic ultrasound, you will perform several exercises focused on rehabilitation of patellar tendinopathy of your knee. They will be resistance training exercises for the lower limbs using weightlifting equipment and elastic bands.

3. Physical tests will be applied at the beginning, middle (4 weeks) and final (after 8 weeks) of the treatment. The tests will be: evaluation of the muscular strength of the gluteus maximus, gluteus medius, quadriceps femoris and triceps sural ("calves") through a portable equipment (dynamometer). You will perform vigorous isometric (sustained) contractions of the lower limbs with sufficient rest interval between the two attempts. The tests will be performed after heating the muscles, which makes the risk of muscular injuries practically nonexistent.

4. To evaluate the motor function, some functional tests (step down, descent of degree by jumping with the two lower limbs and with one lower limb at a time and frontal jumps with one lower limb at a time) will be realized, where an equipment (two cameras connected to a computer program) will record the patterns and possible biomechanical compensation of their movements. To perform these tests, we will apply (with hypo-allergenic adhesive tape) reflective markers on the skin, at specific anatomical sites. All equipment will be sanitized before use.

5. Questionnaires will be applied to verify their level of physical activity / sport practice (IPAQ-short form), the functional characteristics of their knee with pain in the patellar tendon (VISA-P) and visual analogue scale (VAS) to measure your

pain level. At first we will conduct an interview to record important information about your health. You will then be submitted to the two questionnaires and the pain scale described above. The issues will not cause any embarrassment and we do not want to get information that causes discomfort.

6. In addition, the procedure of collecting the maximum amount of tolerated force in the painful patellar tendon (algometry) - through application of compression in the complainant patellar tendon with a specific instrument for this (algometer) - will be performed, as well as the painful tender tendon temperature, by capturing thermographic photographs. These two procedures will be performed with you in the lying position. Since the collection will be done by a qualified professional (physiotherapist), and algometry can generate a small sensation of discomfort in the patellar tendon.

7. Report: At the end of the treatment period we will deliver your evaluation report with the information acquired in your strength and function tests.

All procedures will be performed at the Federal University of São Paulo - Campus Baixada Santista in the laboratory of the Research Group on Rehabilitation and Electrophysical Agents (GPRAE).

This consent form is being made available in two (2) original copies of equal content; being one way for the researcher and the other for the volunteer of this study.

Risks and discomforts

Therapeutic ultrasound poses no risk to your health, and you will undergo a detailed preliminary evaluation. If you are diagnosed with any risk, you will be advised and will not be included in the research, as well as receive necessary information regarding medical follow-up (if necessary). There may eventually be a tiredness or pain resulting from the effort made, but nothing that compromises your health. Tests or exercises may be interrupted by you if necessary. **If there is any discomfort during the tests or exercises, we promise to take you to the nearest emergency room if this is really necessary.**

Warranties

There is no direct benefit to the participant, since it is a research that aims to establish a methodology that can be used in the future. At any time during the study, the evaluated person can have access to the professionals responsible for the research to clarify any doubts or even to withdraw consent and to stop participating in the study without any prejudice. If any problem or personal injury occurs during or after the procedures to which you will be subjected, you will be guaranteed the right to free treatment in the same place that the program will be performed and you will be entitled to indemnity determined by law.

The researcher responsible is Prof. Dr. Carlos Eduardo Pinfildi who can be found at Rua Silva Jardim, 136 - Vila Mathias - Santos/SP - CEP: 11015-020; Phone (13) 3878-3700. If there is any consideration or doubt about the research ethics, please contact the Research Ethics Committee (CEP) – Rua Prof. Francisco de Castro, n°55 - São Paulo, SP - CEP: 04020-050 - Tel: (11) 5571-1062 e (11) 5539-7162 - E-mail: CEP@unifesp.edu.br.

The information obtained will be analyzed together with other volunteers, not being disclosed the identification of the same. The evaluated will also be entitled to be informed about the partial results of the research. We guarantee the use of research data for exclusively academic purposes.

There are no personal expenses for the evaluated at any stage of the study, including examinations and consultations. There is also no financial compensation related to your participation.

Consent statement

I believe I have been sufficiently informed about the information I read or have read to me describing the study **“HIGH-ENERGY DOSE OF THERAPEUTIC ULTRASOUND IN THE TREATMENT OF PATELLAR TENDINOPATHY: A RANDOMIZED PLACEBO-CONTROLLED CLINICAL TRIAL”**

It was clear to me what the purposes of the study were, the procedures to be carried out, their discomforts and risks, the guarantees of confidentiality and permanent clarification. It is also clear that my participation is free of expenses. I voluntarily agree to participate in this study and may withdraw my consent at any time, before or during it, without penalty or loss or loss of any benefit I may have acquired, or in my attendance at this Service.

____

Volunteer Signature / Legal Representative Date___/___/______

____

Name of volunteer / legal representative

____

Signature of witness * Date___/___/______

____

Name of witness

* For cases of patients illiterate and semi-illiterate

____

(only for the person in charge of the project)

I declare that I have obtained in an appropriate and voluntary manner the Free and

Informed Consent of this volunteer for participation in this study.

____

Signature of the person in charge of the study   Date___/___/______

____

Name of person in charge of the study
